# Biomechanical comparison of all-on-4 and all-on-5 implant-supported prostheses with alteration of anterior-posterior spread: a three-dimensional finite element analysis

**DOI:** 10.3389/fbioe.2023.1187504

**Published:** 2023-06-16

**Authors:** Xin Sun, Kangjie Cheng, Yunfeng Liu, Sipeng Ke, Wentao Zhang, Linhong Wang, Fan Yang

**Affiliations:** ^1^ Department of Stomatology, Zhejiang Chinese Medical University, Hangzhou, Zhejiang, China; ^2^ College of Mechanical Engineering, Zhejiang University of Technology, Hangzhou, China; ^3^ Key Laboratory of Special Purpose Equipment and Advanced Processing Technology, Ministry of Education and Zhejiang Province, Zhejiang University of Technology, Hangzhou, China; ^4^ National International Joint Research Center of Special Purpose Equipment and Advanced Processing Technology, Zhejiang University of Technology, Hangzhou, China; ^5^ Center for Plastic and Reconstructive Surgery, Department of Stomatology, Zhejiang Provincial People’s Hospital, Affiliated People’s Hospital, Hangzhou Medical College, Hangzhou, Zhejiang, China

**Keywords:** anterior-posterior spread, three-dimensional finite element analysis, all-on-4, all-on-5, biomechanics

## Abstract

**Introduction:** The all-on-4 concept is widely used in clinical practice. However, the biomechanical changes following the alteration of anterior-posterior (AP) spread in all-on-4 implant-supported prostheses have not been extensively studied.

**Methods:** Three-dimensional finite element analysis was used to compare the biomechanical behavior of all-on-4 and all-on-5 implant-supported prostheses with a change in anterior-posterior (AP) spread. A three-dimensional finite element analysis was performed on a geometrical mandible model containing 4 or 5 implants. Four different implant configurations were modeled by varying the angle of inclination of the distal implants (0°and 30°), including all-on-4a, all-on-4b, all-on-5a, and all-on-5b, and a 100 N force was successively applied to the anterior and unilateral posterior teeth to observe and analyze the differences in the biomechanical behavior of each model under the static influence at different position.

**Results:** Adding an anterior implant to the dental arch according to the all-on-4 concept with a distal 30° tilt angle implant exhibited the best biomechanical behavior. However, when the distal implant was implanted axially, there was no significant difference between the all-on-4 and all-on-5 groups.

**Discussion:** In the all-on-5 group, increasing the AP spread with tilted terminal implants showed better biomechanical behavior. It can be concluded that placing an additional implant in the midline of the atrophic edentulous mandible and increasing the AP spread might be beneficial in improving the biomechanical behavior of tilted distal implants.

## 1 Introduction

The all-on-4 procedure with an implant-supported total prosthesis has been widely used in clinical practice for a mandible with severe alveolar ridge resorption (Malo et al., 2003; Reissmann et al., 2017). In such cases, a distally tilted implant can be employed to minimize the cantilever length due to anatomical limitations, with the maximum allowable angle of inclination for the distal implant being 45° (Malo et al., 2003; Malo et al., 2005). Previous studies have reported the incidence of mechanical complications associated with the rehabilitation of the edentulous maxilla and mandible. For example, mechanical complications were reported in 58.8% of provisional prostheses and 7.3% of definitive prostheses over a follow-up period of 5–13 years (Malo et al., 2019), while another study reported a mechanical complication incidence of 36.7% in males undergoing full-arch rehabilitation of the completely edentulous mandible over a follow-up period of 10–18 years (Malo et al., 2019). Another retrospective cohort study with up to 10 years of follow-up reported that 19.8% of patients experienced biological complications, while mechanical complications were reported in 27.1% of cases (Grandi and Signorini, 2021).

The cantilever acts as a force amplifier for implants, abutment screws, prosthesis screws, adhesions, or implant-bone interface while the patient is chewing. This is especially true if the crown is short or if the patient is engaged in para-functional activities. Long cantilevers lead to increased load distribution on the implant, which can lead to biomechanical complications (McAlarney and Stavropoulos, 2000). When cantilever extensions exceed 15 mm, there is a higher risk of encountering mechanical or technical complications (Salvi and Bragger, 2009). Determining the appropriate cantilever length (CL) is crucial as it directly impacts the biomechanical stability and long-term success of the prosthesis.

The ratio of cantilever length to anterior-posterior (AP) spread has been identified as one of the factors associated with the incidence of mechanical complications. The AP spread was first defined by English (English, 1990) as the distance between a line connecting the distal of the most distal implants of a full-arch implant-supported prosthesis and a line through the center of the most anterior implant. A 2-year clinical retrospective study by Drago et al. showed that a CL-AP ratio of 0.5–0.6 during a functional prosthesis generally led to the success of a temporary prosthesis (Drago, 2017). However, another study indicated that using a single CL-AP ratio alone is not necessarily a reliable predictor of cantilever ability (McAlarney and Stavropoulos, 1996). In a study of full-arch clinical cases, McAlarney et al. found that cantilever loading increased the loads distributed to implants, and according to relevant mathematical models, associated clinical complications could be reduced when the AP spread was greater than 11.1 mm (McAlarney and Stavropoulos, 2000). To date, guidelines for the design of frameworks with distal cantilever segments and AP spread have not been established.

Moreover, in cases of the atrophic mandible, the addition of an implant in the anterior region of the jaw has been shown to increase the AP spread (English, 1990). Most studies have focused on four, six, or eight implants, and few have investigated the effect of five implants on the biomechanics of the mandible. Bhering et al. found that the all-on-6 implant configuration exhibits more favorable biomechanical behavior compared to the all-on-4 configurations using three-dimensional finite element analysis (Bhering et al., 2016). Similar results of higher forces with decreasing number of supporting implants have been reported in an *in vivo* study (Duyck et al., 2000). A randomized controlled clinical trials showed no significant difference in the 5-year implant failure rate and marginal bone absorption between all-on-4 and all-on-6 (Tallarico et al., 2016), and the same result was reported in a recent 3-13-years retrospective cohort study (Zhang et al., 2022).

So far, the extent of the difference in loading between vertically placed and angled implants in the presence of abundant distal bone remains unclear. In the case of edentulous mandibles, the addition of a mandibular midline implant to increase the AP spread without changing the cantilever length raises questions regarding the overall force changes between groups, which have yet to be investigated.

Therefore, the objective of this study was to use three-dimensional finite element analysis to evaluate the changes in the prosthesis, implant, and bone stresses resulting from the addition of a fifth implant in the anteromedial region of the dental arch with either increased AP spread or modified implant tilt angle, while maintaining a fixed cantilever length. The null hypothesis of the study was that there would be no difference in biomechanical behavior between the four and five implant configurations in an atrophic mandible following the addition of AP spread.

## 2 Materials and methods

**FIGURE 1:**
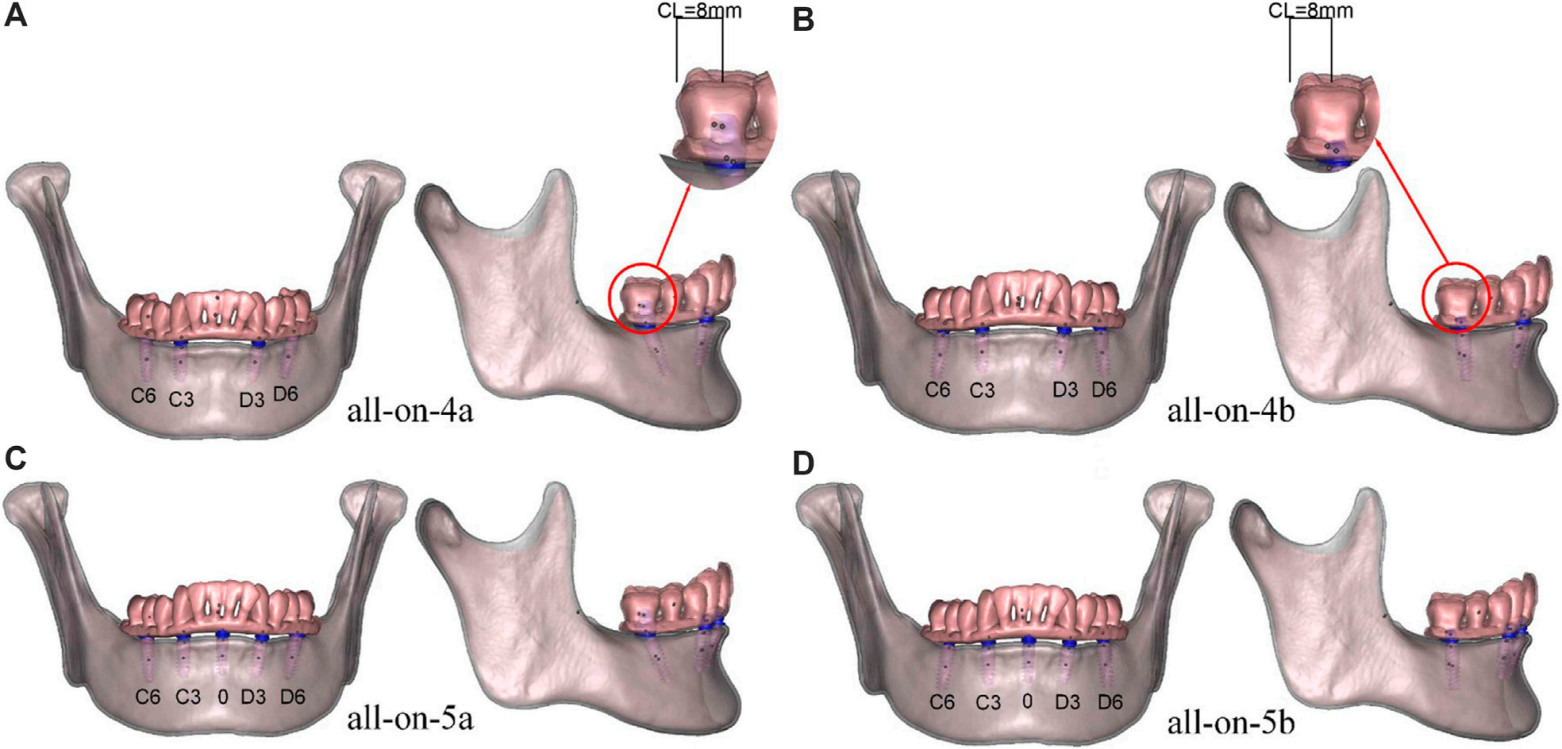
**(A)** all-on-4a model, **(B)** all-on-4b model, **(C)** all-on-5a model, **(D)** all-on-5b model, the numbers C6 to D6 represent the dental positions in each model. The cantilever length shown on the models was 8 mm.

A finite element model of the edentulous mandible was constructed from cone beam computed tomography (CBCT) data, and 4 or 5 implants were placed in the mandible (Figure 1). Occlusal loading was simulated in the models to analyze the biomechanical behavior of the components and bone tissue.

### 2.1 Design of the components

In this study, mandible CBCT data were acquired from a 30-year-old healthy female volunteer using a NewTom3G scanner (Cefla Dental Group, Imola, Italy) with exposure settings of 110 kV, 6.0 mA, 9.0-s exposure time, and a voxel size of 0.027 mm^3^. The study protocol was approved by the Ethics Committee of Zhejiang Provincial People’s Hospital (No.QT2022093), and written informed consent was obtained in advance. The images are required to be clear, and the imaging data show no craniomaxillofacial developmental deformities, no periodontal disease or jaw lesions, and no obvious crowding of the dentition. The CBCT data were imported in Digital Imaging and Communications in Medicine (DICOM) format into Mimics software (V21.0; Materialise), and a rough 3D contour of the mandible was obtained by thresholding and masking. The resulting file was exported in STL format and further processed in Magics software (V20.03; Materialise) to segment the dental crown and mandible, resulting in models of the dentition and mandible. The models were imported into Geomagic Studio software (V12; Geomagic) for refinement, where a three-dimensional solid model of the mandible was accurately calculated to obtain a complete and smooth surface. The model was simplified to a cortical bone shell with a uniform thickness of 1.5 mm, and the cancellous bone structure was obtained using the total offset command. The cortical and cancellous bone models were exported in Stereolithography (STL) format. Finally, a prosthesis and titanium frame model was obtained by extraction of the dentition, and the prosthesis was restored to the occlusal surface of the bilateral first molars.

The implants in the model refer to the Nobel active system with 3.5 mm × 11.5 mm; 4.3 mm × 11.5 mm; 4.3 mm × 13 mm implants. The implants were categorized into three types based on their length and placement: 0-point implants were 3.5 mm × 11.5 mm, 3-point implants were 4.3 mm × 11.5 mm and 6-point implants were 4.3 mm × 13 mm, representing the central, canine, and first molar sites, respectively. Two abutment designs were employed, including a straight abutment with a height of 3.5 mm and an angle of 0°, and a 30° angled abutment with a height of 4.5 mm. Models were designed in SolidWorks (V2014; Dassault Systemes) based on the implant shape data, abutments, and screws (Table 1).

**TABLE 1:** Implant and abutment characteristics, Element and node numbers of models of each group.

Groups	Implant number	Anterior implant				Posterior implant				Number of elements	Number of nodes
		Location	Angle	Implants	Abutments	Location	Angle	Implants	Abutments		
All-on-4a	4	2-3 point	0°	2–4.3 × 11.5 mm	0°	2-6 point	30°	2–4.3 × 13 mm	30°	2818252	605681
All-on-4b	4	2-3 point	0°	2–4.3 × 11.5 mm	0°	2-6 point	0°	2–4.3 × 13 mm	0°	2185367	483237
All-on-5a	5	2-3 point	0°	2–4.3 × 11.5 mm	0°	2-6 point	30°	2–4.3 × 13 mm	30°	2888508	611200
		1-0 point		1–3.5 × 11.5 mm							
All-on-5b	5	2-3 point	0°	2–4.3 × 11.5 mm	0°	2-6 point	0°	2–4.3 × 13 mm	0°	2784221	597829
		1-0 point		1–3.5 × 11.5 mm							

### 2.2 Grouping of models

**FIGURE 2:**
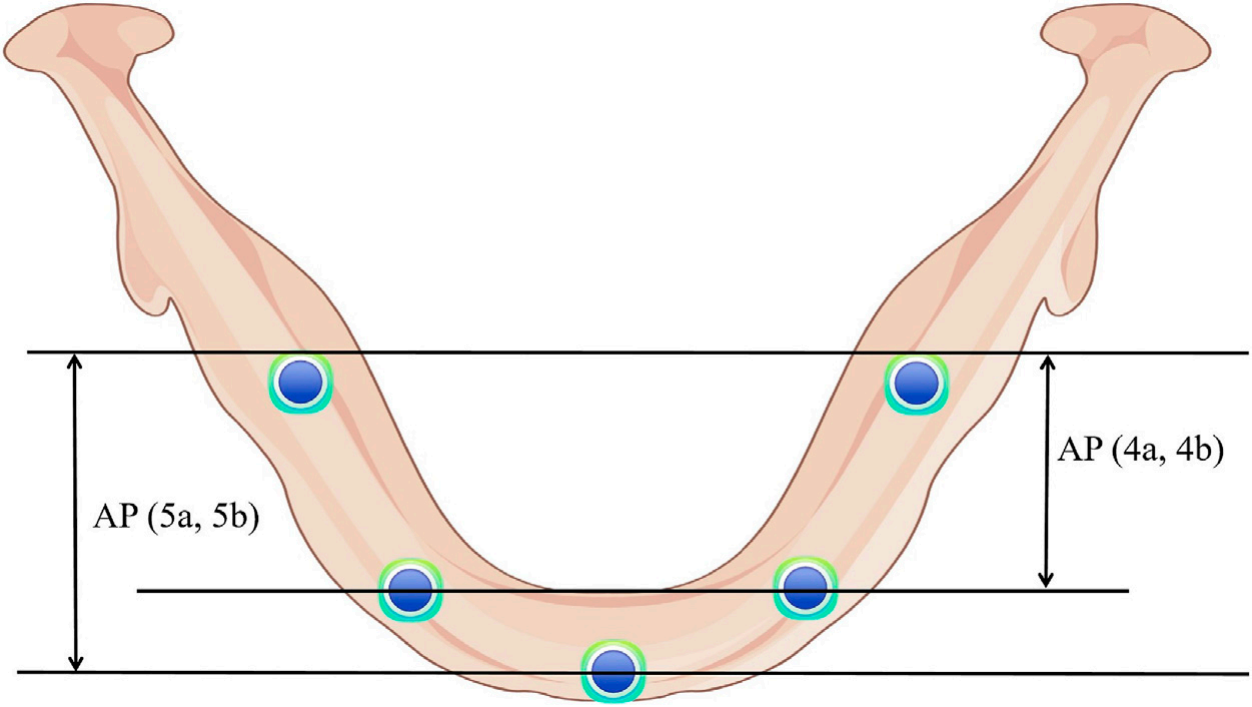
Anterior-posterior (AP) spread of each group.

The models were grouped based on the placement of four or five implants in the edentulous mandible. Four groups were established: all-on-4a, all-on-4b, all-on-5a, and all-on-5b (Figure 1), with AP spreads of 18.5 mm (all-on-4a, all-on-4b) and 22.5 mm (all-on-5a, all-on-5b) (Figure 2). The dental positions were numbered from the right to the left side of the mandible: C6, C3, 0, D3, D6, with C represents the right region, D represents the left region, and 0 represents the median position (Figure 1). The bilateral terminal implant sites were located at C6 and D6, with a cantilever of 8.0 mm (Figure 2).

### 2.3 Metrics, meshing and material properties

The Abaqus software (V6.13; Dassault Systemes) was utilized to calculate displacements of the implants and superstructures, strains in the peri-implant bone and von Mises stresses. Strains in the peri-implant bone can be used to predict bone remodeling properties compared to the bone resorption threshold (Frost, 1992; Frost, 2001; Frost, 2004; Sugiura et al., 2000). Maximum von Mises stresses were recorded on the peri-implant bone, implants and superstructure to describe the material deformation stress state (Mellal et al., 2004). As the geometry of the mandible model is very complex, this study used tetrahedral elements of arbitrary geometries of higher simulation accuracy and computational cost to mesh the mandibular models for subsequent numerical calculations. The mandible was hypothesized to be isotropic, each component of the model was assigned a value based on the mechanical properties in the relevant literature in Table 2 (Macedo et al., 2017; Ozan and Kurtulmus-Yilmaz, 2018; Liu et al., 2019; Zhang et al., 2022).

**TABLE 2:** Mechanical properties of the materials.

Materials	Elastic modulus (MPa)	Poisson ratio
Cortical bone	13700	0.3
Cancellous bone	1370	0.3
Acrylic material	3000	0.35
Titanium material	112000	0.35

### 2.4 Constraints and loading conditions

**FIGURE 3:**
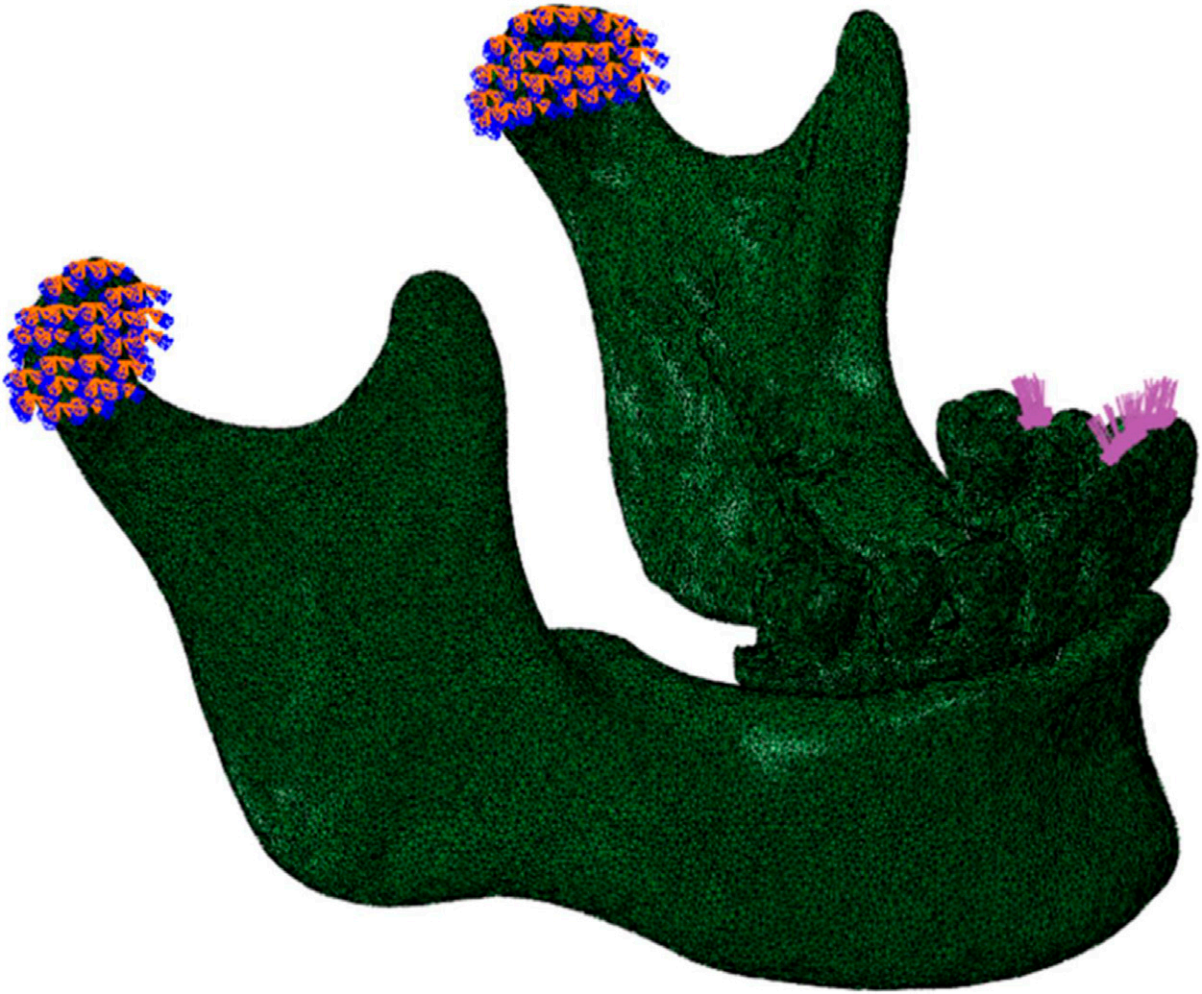
Lateral view of the mandible of the finite element model, showing constraints and loads. The restraint was located at bilateral condyles. A 100 N static load was applied vertically to the anterior (central and lateral incisors) and the left posterior (second premolar and first molar) regions.

To accurately simulate anatomically normal mandibular function, the bilateral condyles were assumed to be locked in the articular fossa and constrained to full degrees of freedom (Figure 3) (Cheng et al., 2020; Orassi et al., 2021; Orassi et al., 2021). Materials were modeled with small deformations and linear elastic behavior. The implant-bone interface was set to be 100% contact, with rigid restraints to prevent relative sliding during application. Two loading modes were employed for each model. To simulate the anterior and unilateral posterior chewing forces, a static load of 100 N was applied, distributed evenly over a 4 mm^2^ oval area. The static load magnitude remained constant throughout the simulation. In the anterior region, the central and lateral incisors received vertical loading (A loads), while in the posterior region, the left second premolar and first molar were subjected to vertical loading (P loads) (Figure 3).

## 3 Results

### 3.1 Displacement

The displacement values of each component in the four groups are shown in Tables 3, 4. The 5-implant groups showed less displacement than the 4-implant groups when the distal implants were tilted, regardless of the load position. However, the 5-implant groups showed increased displacement when the distal implants were placed axially. The distal tilted groups (all-on-4a and all-on-5a) showed less displacement than the axial groups (all-on-4b, all-on-5b), regardless of the number of implants. The maximum displacement of the implants occurred at the apical region, whereas that of the abutments and screws occurred in the cervical and bottom regions. Concerning the superstructure, the most significant displacement was observed in the lower left lateral incisor. The direction of displacement of the model was consistent with the direction of loading.

**TABLE 3:** Displacement (mm) —— Anterior.

Components	Implants					Abutment					Prosthetic screw					Titanium frame	Restoration
Number	C6	C3	0	D3	D6	C6	C3	0	D3	D6	C6	C3	0	D3	D6		
All-on-4a	0.54	0.60	—	0.61	0.54	0.50	0.59	—	0.59	0.49	0.44	0.57	—	0.59	0.42	0.61	0.62
All-on-4b	0.56	0.65	—	0.66	0.55	0.52	0.64	—	0.64	0.51	0.49	0.62	—	0.63	0.48	0.67	0.57
All-on-5a	0.46	0.51	0.53	0.52	0.46	0.42	0.50	0.52	0.50	0.42	0.37	0.49	0.51	0.49	0.37	0.52	0.52
All-on-5b	0.60	0.69	0.71	0.70	0.59	0.56	0.67	0.70	0.68	0.54	0.52	0.66	0.69	0.67	0.51	0.70	0.71

**TABLE 4:** Displacement (mm) —— Posterior.

Components	Implants					Abutment					Prosthetic screw					Titanium frame	Restoration
Number	C6	C3	0	D3	D6	C6	C3	0	D3	D6	C6	C3	0	D3	D6		
All-on-4a	0.36	0.41	—	0.42	0.38	0.33	0.40	—	0.42	0.35	0.29	0.39	—	0.41	0.31	0.43	0.43
All-on-4b	0.35	0.41	—	0.43	0.37	0.32	0.40	—	0.42	0.34	0.30	0.39	—	0.41	0.32	0.44	0.45
All-on-5a	0.31	0.35	0.37	0.37	0.33	0.28	0.34	0.36	0.36	0.30	0.25	0.33	0.36	0.35	0.27	0.37	0.37
All-on-5b	0.39	0.46	0.48	0.48	0.41	0.36	0.45	0.47	0.47	0.38	0.34	0.44	0.47	0.46	0.35	0.48	0.48

### 3.2 Strains at the site of cortical and cancellous bone

Finite element results showed that the maximum principal strain was all located in the bone tissue near the neck of the implant. Therefore, the top ten maximum principal strain values of the cortical and cancellous bone parts around each group of implants were selected for statistical analysis, representing the modeling and remodeling behavior of the bone tissue around the neck of the implant. The normality of the data distribution was assessed using the Shapiro-Wilk test. Group comparisons were performed using one-way ANOVA followed by Tukey’s test. IBM SPSS version 22.0 (SPSS Statistics 2.0, SPSS, Chicago, IL, United States) was used for statistical analysis, and statistical significance was set at 0.05.

**FIGURE 4:**
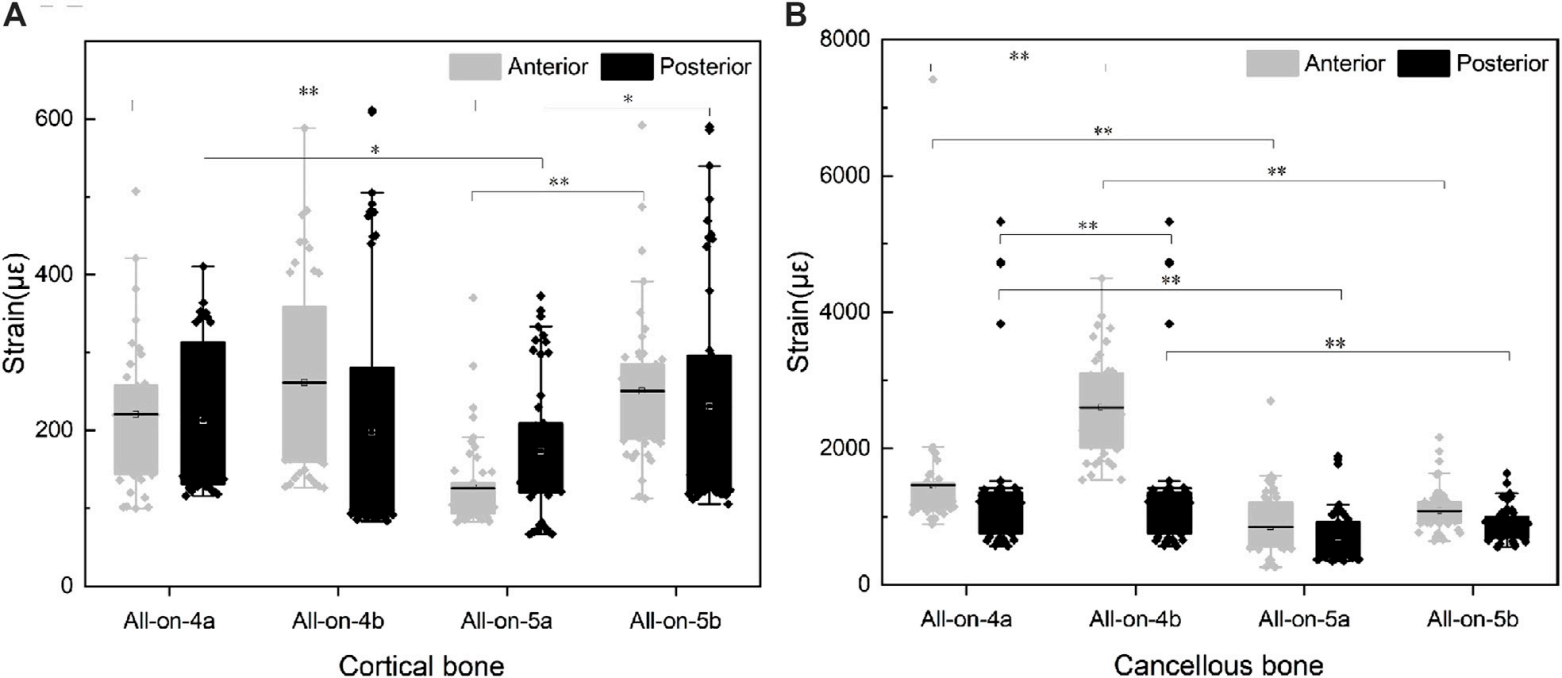
Strain in the cortical bone **(A)** and cancellous bone **(B)** around implants.

Regarding the effect of the number of implants on the strain of the bone tissue, in the cortical bone, the strain in the all-on-5a group was lower than that in the all-on-4a group (*p* < 0.05), but the difference between the all-on-4b and all-on-5b groups was not significant (*p* > 0.05) (Figure 4A). The difference in strain on the cancellous bone between the 5-implant and the 4-implant models was significant whether the implant was placed at a 0° or 30° angle (p < 0.05) (Figure 4B).

Regarding the effect of the distal implant angle on bone tissue strain, the strain on the cortical bone surface of the model in the all-on-4a the group was not significantly different from that in the all-on-4b group (*p* > 0.05), regardless of whether the load was applied to the anterior or unilateral molar region. The strain on the cortical bone surface in the all-on-5a group was significantly lower than that in the all-on-5b group (*p* < 0.05). In contrast, the *p*-value results for the strain on the cancellous bone surface were opposite to those observed on the cortical bone (Figure 4B).

### 3.3 Von Mises stress assessments

#### 3.3.1 Von Mises stress of implants

**FIGURE 5:**
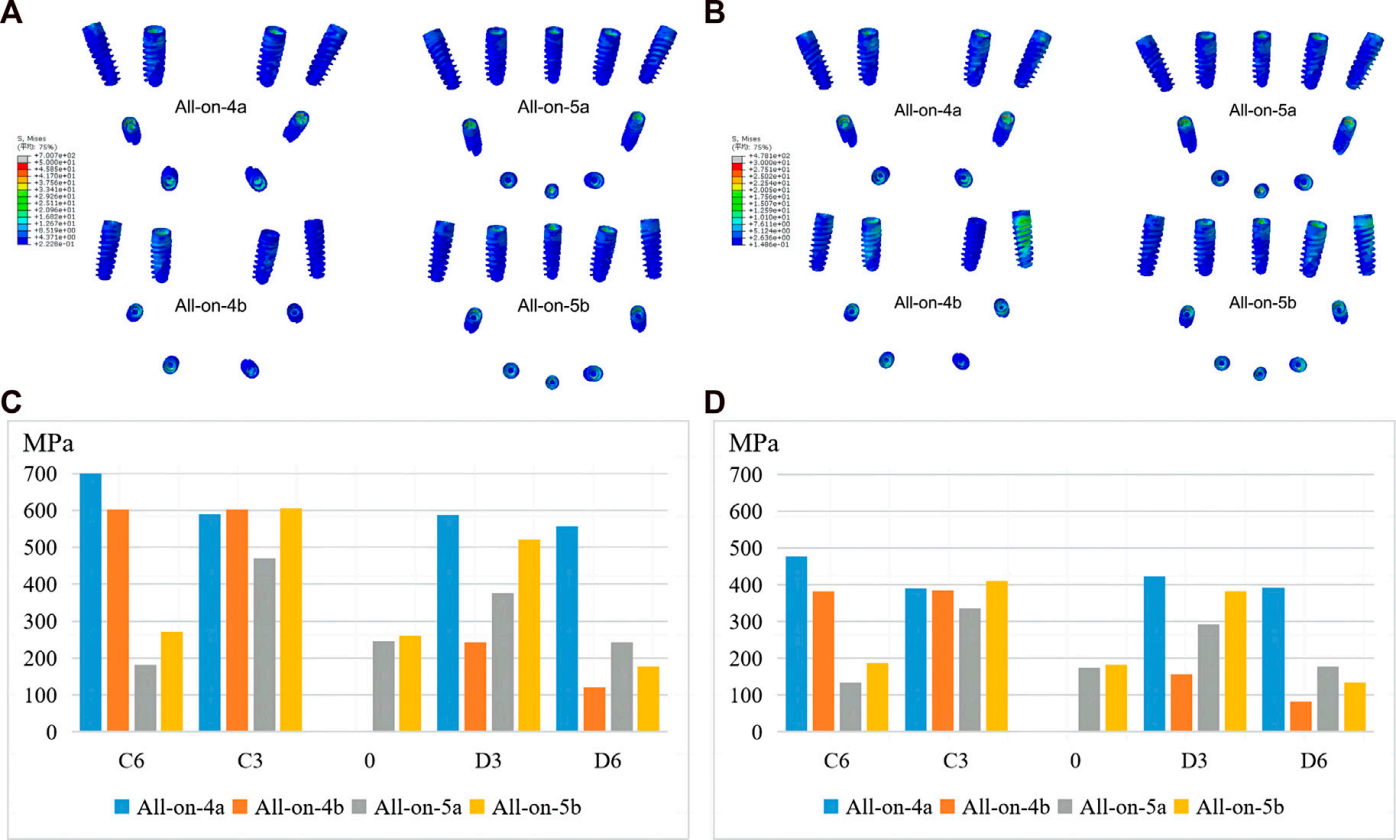
Maximum von Mises stresses of implants under anterior **(A, C)** and posterior loading **(B, D)**.

Irrespective of whether the loads were in the anterior or posterior region, the all-on-5a group showed significantly lower stress values when the distal implants were tilted, especially for implants located in the D6 and C6 sites, the stresses were reduced by 56.4% and 74.1% for loads located in the anterior region (A loads) and by 54.6% and 72.0% for loads in the posterior region (P loads). However, when the distal implants were vertical, the stress distribution of the implants in the all-on-5b group was not significantly reduced. When the same number of implants, the all-on-4b group had a lower overall von Mises stress level than the all-on-4a group, whereas the all-on-5b group had a higher stress level (Figure 5). The highest von Mises stress concentration occurred in two locations: the implant-bone interface near the annular region of cortical bone and the medial side of the implant attached to the abutment (Figure 5). Increasing the AP spread resulted in a decrease of maximum von Mises stress of implants in the tilted groups.

#### 3.3.2 Von Mises stress of cortical and cancellous bone

The results showed that compared to the all-on-4a group, the maximum von Mises stress in the cortical bone was significantly reduced in the all-on-5a group, especially when loaded in the anterior region. However, the maximum von Mises stress in cortical bone increased in the all-on-5b group when the terminal implants were placed axially. In addition, the maximum von Mises stress in the cancellous bone was lower in the 5-implant groups than in the 4-implant groups under both anterior and posterior loading conditions (Table 5). The maximum von Mises stress in the alveolar bone around the implant in each model group was found to be located in the cortical bone region near the implant neck.

**TABLE 5:** Maximum von Mises Stress (MPa)- Cortical Bone and Cancellous Bone.

Load position	Anterior					Posterior				
Number	C6	C3	0	D3	D6	C6	C3	0	D3	D6
All-on-4a	6.18 | 8.94	5.32 | 2.44	—	9.67 | 3.42	6.78 | 2.69	5.75 | 5.80	2.99 | 1.66	—	10.29 | 2.38	4.45 | 6.49
All-on-4b	7.95 | 6.16	5.95 | 5.45	—	6.65 | 7.81	4.31 | 5.11	8.30 | 3.86	4.27 | 3.50	—	3.84 | 5.13	3.96 | 4.85
All-on-5a	2.62 | 4.81	5.98 | 1.77	4.22 | 1.05	10.84 | 1.56	3.14 | 4.61	5.02 | 1.50	4.07 | 1.21	3.16 | 1.07	8.81 | 0.98	3.28 | 3.88
All-on-5b	6.51 | 2.59	6.17 | 2.64	8.14 | 3.33	11.63 | 2.00	8.50 | 2.60	8.05 | 2.09	3.80 | 1.73	5.17 | 2.34	11.05 | 1.33	5.04 | 1.91

#### 3.3.3 Von Mises stress of the superstructure

Under load P, the maximum von Mises stress in the superstructure was lower than under load A, and the stresses were mainly concentrated in the titanium frame. Notably, the maximum stress was significantly reduced in the 5-implant groups compared to the 4-implant groups, particularly in the distal implant vertical placement groups (Table 6).

**TABLE 6:** Maximum von Mises stress (MPa) —— Superstructure.

Load position	Titanium frame—Anterior	Titanium frame– Posterior	Restoration– Anterior	Restoration– Posterior
All-on-4a	563.12	415.42	28.13	17.71
All-on-4b	518.66	305.19	47.24	28.76
All-on-5a	388.21	280.28	23.35	15.68
All-on-5b	179.38	118.58	33.65	23.45

#### 3.3.4 Von Mises stress of abutments and screws

**FIGURE 6:**
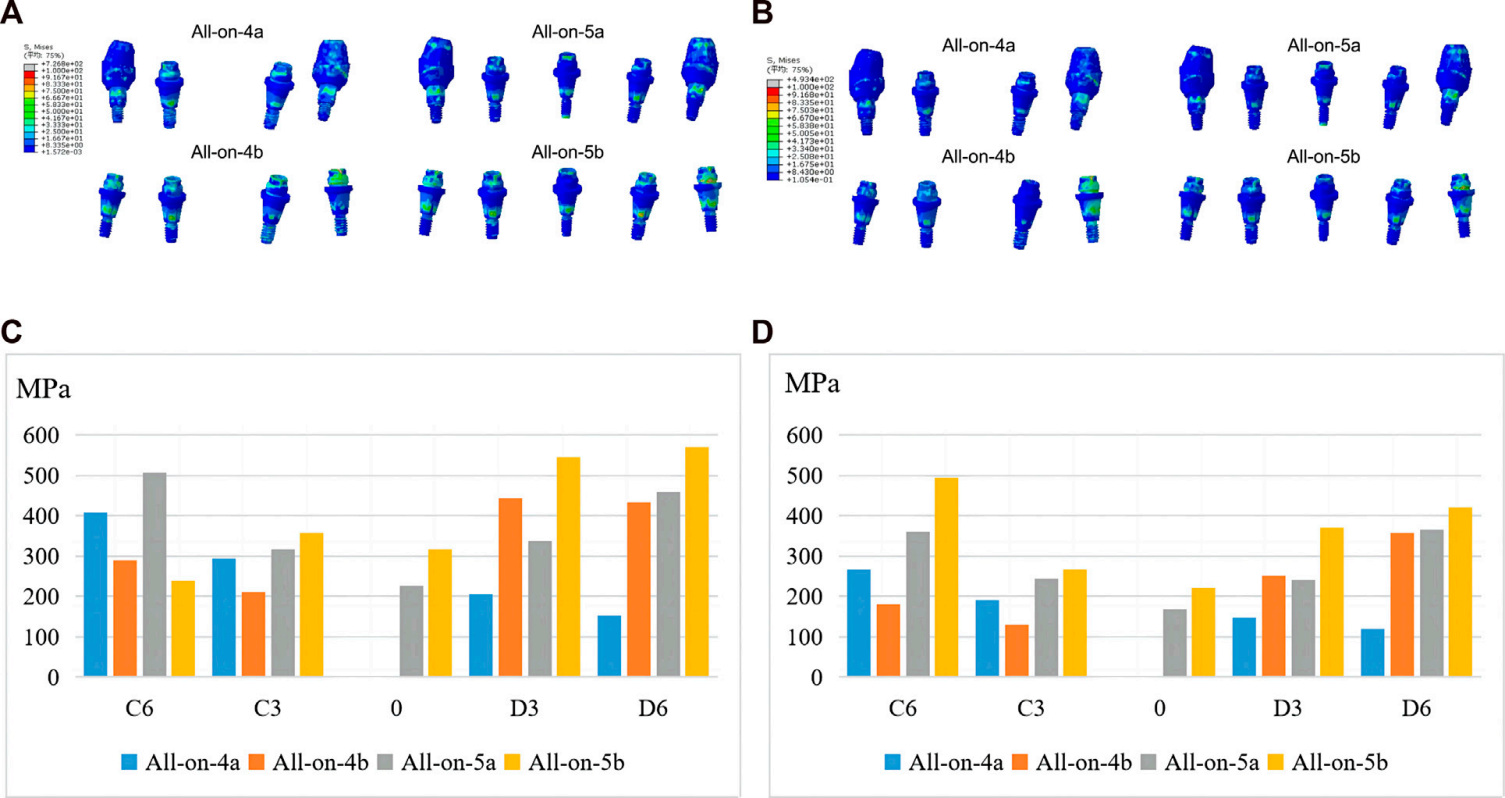
Maximum von Mises stresses of abutments under anterior **(A, C)** and posterior loading **(B, D)**.

**FIGURE 7:**
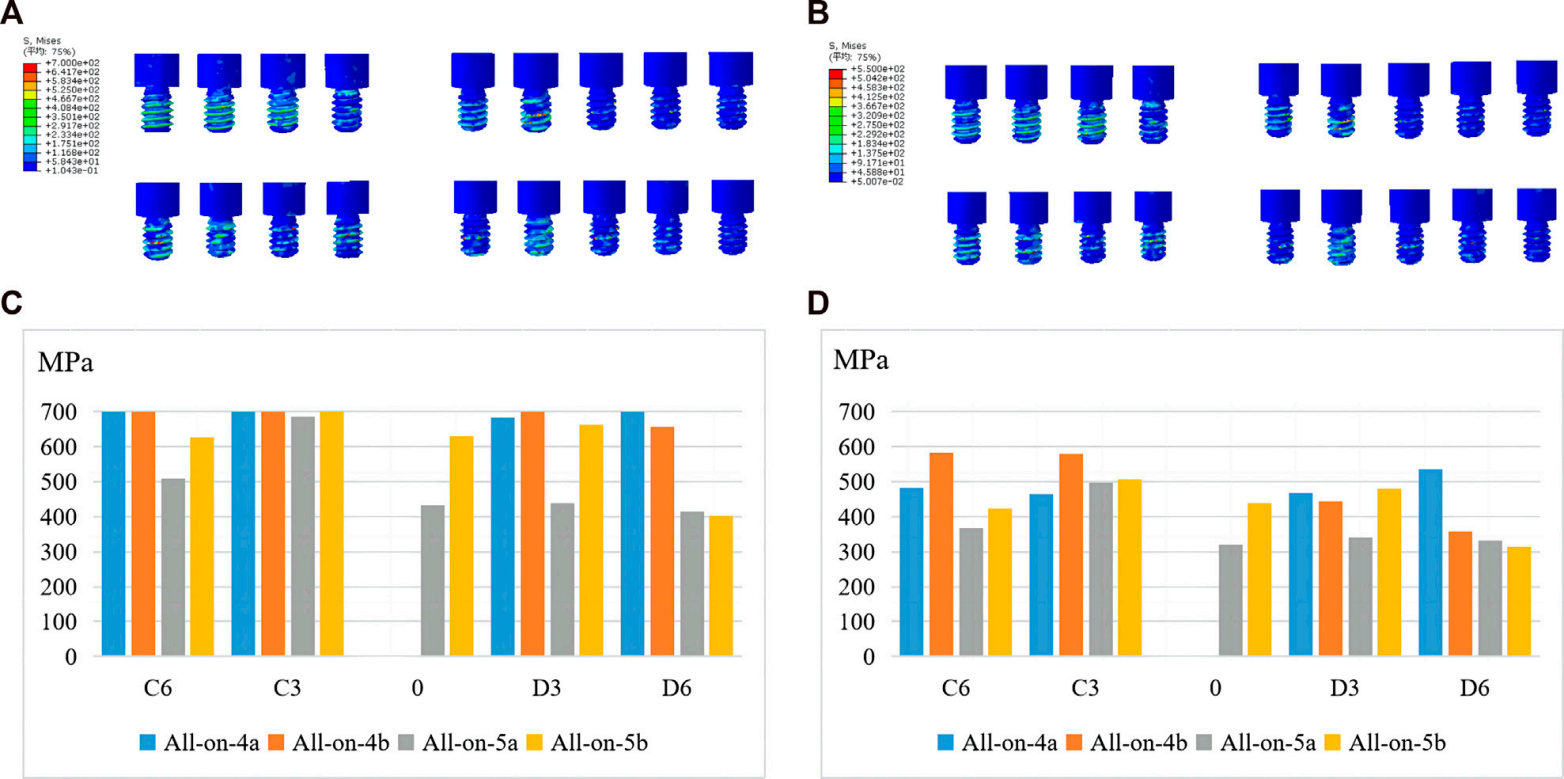
Maximum von Mises stresses of screws under anterior **(A, C)** and posterior loading **(B, D)**.

In terms of stress distribution in the abutments, the maximum von Mises stress increased in the all-on-5 groups compared to the all-on-4 groups, regardless of whether the distal implants were vertical or inclined (Figure 6). The peak stress was concentrated in the ring region in contact with the titanium frame and above the threaded portion in contact with the implant (Figure 6). In addition, the maximum von Mises stress often occurred in prosthetic screws, particularly at the D6, C3, and C6 sites, suggesting that screws at these sites are more prone to mechanical complications. The results also showed that the maximum von Mises on prosthetic screws was reduced in the all-on-5 groups compared to the all-on-4 groups (Figure 7). The distribution of maximum von Mises stress on prosthetic screws was observed at the neck thread of the screws in all models (Figure 7).

## 4 Discussion

Finite element analysis has been widely used to detect the biomechanical behavior of functional bite force in prosthetics and surrounding tissues. Due to the complex structure of bone, some assumptions were often made to simulate the actual situation, so that the accuracy of the model was the key to the experiment. In this study, the mandible model was obtained from a patient eligible for CT tomography, and implants and abutments were generated in the software according to actual parameters, which was consistent with clinical practice. Previous studies have suggested that the success rate of 3D finite element mechanical analysis was related to the number of elements and nodes in the digital model (DeTolla et al., 2000). In the digital entity model constructed in this study, the number of elements and nodes exceeded 79 000 and 150 000 respectively, which was sufficient to maximize the acuity of mechanical analysis compared with similar studies (Fanuscu et al., 2004; Koca et al., 2005). Although biomechanical studies have shown that mandible is characterized by significant orthogonal anisotropy and non-homogeneous material (Schwartz-Dabney and Dechow, 2003), this study focused on the biomechanical differences of implant and superstructure between the groups. The orthogonal anisotropy simulation mainly affected the stress distribution of the implant-bone interface and had no significant effect on the stress values of the implant, abutment, and superstructure. To streamline the calculation of experimental findings, the mandible was presumed to possess isotropic characteristics in accordance with the prevailing approach adopted in previous studies (Macedo et al., 2017; Ozan and Kurtulmus-Yilmaz, 2018; Liu et al., 2019; Zhang et al., 2022; Cheng et al., 2023). Experimental strain gauge and theoretical stress analysis methods have demonstrated that assuming materials are linear, elastic, and isotropic results in an agreement between experimental and theoretical results (Huiskes, 1982).

Anterior-posterior (AP) spread assessments have been commonly used to determine the length of a distal cantilever that can be extended from an implant-supported fixed full-arch prosthesis. However, recommendations based on AP spread assessments to calculate cantilever lengths have not been validated by prospective scientific evaluations (Walter and Greenstein, 2020). To date, no studies have focused on the influence of increasing the AP spread by adding an implant in the anterior mandible on stress distribution. In this study, three-dimensional finite element models of edentulous mandibular fixed restorations supported by 4 or 5 implants were constructed and loaded vertically and statically to analyze the effects of the two different AP spreads and implants angle on displacement, strain, and stress in four different models. The results showed that increasing the AP spread by adding an implant in the anterior median of the mandible is beneficial to improve the stress distribution when the all-on-4 distal implant is inclined. However, when the distal implant is vertical, such an operation appears to be insignificant from a three-dimensional finite element perspective. Therefore, the null hypothesis that there is no difference in biomechanical behavior between 4 and 5 implants in an atrophic mandible was partially rejected.

In the present study, to analyze the experimental results more conveniently, the variables were controlled, and the placement and position parameters of each implant group were standardized so that the cantilever length of each group was the same. Studies have shown that cantilever length is an important factor in increasing peak bone stress in the fixed implant restoration in edentulous jaws (Bhering et al., 2016; Saleh et al., 2015). Currently, cantilever lengths of less than 10.0 mm are recommended in clinical practice (Correa et al., 2012; Shackleton et al., 1994). In this study, the measured cantilever length was in this range (8.0 mm). Edentulous arches are divided into three types according to their geometric shape: ovoid, tapered, and square. Ovoid arches are the most common, followed by square and then tapered, a tapered or ovoid arch shape allows for a more favorable AP spread of implants than a square arch, while a square arch results in implants being placed in a straight line, and therefore has a shorter AP spread (Celebi et al., 2016). Arch form is one of the factors that should be considered when planning the size of a distal cantilever of a full-arch prosthesis as it affects the AP spread.

In this study, it was observed that the all-on-5a group resulted in lesser displacement in various aspects such as implants, abutments, prosthetic screws and superstructure compared to the all-on-4a group. Conversely, the total displacement of all-on-5b was greater than that of all-on-4b with axial placement of the distal implants. These results suggest that the all-on-5 technique may improve the initial implant stability in cases where the distal implants were tilted, thereby increasing the implant success rate. Furthermore, there was no evidence of instability due to stress imbalance between the two groups of implants. However, these advantages were not apparent when the distal implants were placed vertically.

Furthermore, strain is an important factor in determining the behavior of bone remodeling (Frost, 2001), and it has been recognized that the implant-bone complex should be stressed within a certain range for physiological homeostasis and optimum functioning, with intraosseous strains ranging from 100 to 1500 με, When the strain is in the 1500 με to 3000 με range, the bone becomes slightly overloaded and will be remodeled to repair the damage and eventually acclimate to the load (Frost, 1983; Frost, 1990). In this experiment, the strain of cortical bone at individual sites was less than 100 με, showing a state of bone resorption, suggesting that cortical bone at the implant margin is prone to resorption, which is consistent with clinical practice. The strains of cancellous bone around all implants were located in the interval of 100 με∼3000 με and the osseointegration of the implant and surrounding cancellous bone could be achieved better under the stimulation of occlusal force during the bone healing period. However, due to the load of 100 N in this experiment, the strain of cancellous bone at individual sites in all-on-4 groups were close to 3000 με. If the applied load became larger, the strain would exceed 3000 με, and the bone tissue was damaged and in the range of pathological bone remodeling, which was unfavorable to bone healing compared with all-on-5. Therefore, it is necessary to optimize the number of implants to reduce the resorption of the surrounding bone.

Within the limited mandibular arch, a significant change in AP distance is not possible and only increased by 4 mm in this study. The stress distribution on the implants in the all-on-5a group was lower than that in the all-on-4a group, especially at the distal implant, and the stress distribution in the all-on-4b group was even lower than that in the all-on-5b group, except at the D6 position. This suggests that the addition of the AP spread in the all-on-5 approach has better biomechanical behavior when the distal implant is placed in an oblique position. The maximum von Mises stress of the implant was located at the implant-abutment junction, and a small stress was located between the implant neck and cortical bone. It is believed that this junction is the weakest area of the implant and is prone to complications such as stress fatigue and mechanical fracture. Nedir et al. (2006) also suggested that implant fracture mainly occurs at the implant-abutment junction. Similar stress distrubition results were found in the cortical and cancellous bone. The stress phogram showed that the total stress values at the bone surface of the all-on-5a model were lower than those of the all-on-4a model, the stresses in the cancellous bone were lower than those in the cortical bone, and the peak von Mises stressed of all the mandibular models occurred in the cortical bone adjacent to the implant neck. This result was also consistent with several previous studies (Gumrukcu and Korkmaz, 2018; Saleh et al., 2015). Therefore, this region was most likely to cause stress overload, suggesting that the stress concentration in the cortical bone is closely related to the bone level at the implant margin. It can be concluded that when the all-on-4 distal implants are placed at an oblique angle, placing an implant in the anterior part of the mandible and adding the AP spread can help reduce the stresses on the bone tissue.

Furthermore, the stress distribution on the prosthetic abutment, screws and superstructure was evaluated, with the maximum stress found at the neck thread (Figure 7), suggesting a higher probability of mechanical complications such as screw loosening and fracture. This finding was consistent with a previous clinical study (Sanchez-Torres et al., 2021). The study also found that the all-on-5 approach resulted in significantly less stress on the titanium framework, especially when the terminal implants were vertical, suggesting that the addition of an extra implant in the anterior mandible could reduce mechanical complications. Due to the difference in modulus of elasticity, the titanium frame was subjected to greater stress relative to restorations. Regardless of whether the distal implants were tilted or vertical, the all-on-5 groups had significantly less stress on the titanium frame, especially when the terminal implants were vertical (−65.41% and −61.15%), which may be related to the difference in the direction of occlusal force transmission, suggesting that stresses on the superstructure can be shared in the clinic by adding an implant in the anterior part of the mandible to reduce mechanical complications.

In conclusion, according to the results of displacement, strain, stress on bone tissue and implant, AP spread was increased by adding an implant to the anteromedial aspect of the mandible, when the distal implant was tilted, that is, the all-on-5a group showed a more dominant biomechanical behavior. We speculated that this might be because, under the same load, when the distal implant was tilted, unlike when the single implant was implanted, part of the stress transmitted from the superstructure to the implant was dispersed by the bone tissue, resulting in less stress on the implant than when the vertical implant was implanted. In addition, the all-on-5a group added implants anteriorly, increasing the AP spread, and all implants formed a triangular structure, which was more stable compared to all-on-4a and, therefore better shared the stresses.

One limitation of this study is that, due to the nature of the model chosen, the prostheses were designed to mimic their original positions, resulting in relatively short cantilever lengths in all groups, and due to the shape of the dental arch, limiting the increase in AP spread. Therefore, the degree of influence between AP spread and cantilever was not very obvious. Although the AP spread of all-on-5 was only increased by 4 mm, the results showed that the same length of the cantilever and a shorter increase in AP spread could help improve the biomechanical behavior of the superstructure and improve the success rate of implantation surgery. The study was also limited by the use of static rather than dynamic forces. To address these limitations, we recommend future research investigate different dental arch shapes, cantilever lengths, and AP spread to further our understanding in this area.

However, due to a large number of theoretical assumptions in the simulation process, and a series of factors such as structural simplification and material property uncertainty, there are inevitable errors between the simulation and the actual structure. The minimum mesh size of this experimental model was 0.1 mm, and the discrete error was controlled within 3%. According to the Guide for Verification and Validation in Computational Solid Mechanics outlined by the American Society of Mechanical Engineering (ASME), it is deemed imperative to undertake subsequent experimental investigations aimed at validation. These experimental endeavors are crucial in ascertaining the accuracy and reliability of the computational model. In addition, given the limitations of existing finite element models and the complexity of masticatory biomechanics, future studies such as clinical trials may help quantify and understand the long-term effects of all-on-5 and demonstrate the potential value of all-on-5.

## 5 Conclusion

Within the limitations of this study, it can be concluded that increasing AP spread by adding an extra implant in the mandibular midline appears to be beneficial in improving the biomechanical behavior of all-on-4 groups with tilted distal implants, while no improvement was observed in distal axial groups. AP spread is not the only aspect to consider when designing an all-on-4 fixed full-arch implant-supported prosthesis.

## Data Availability

The original contributions presented in the study are included in the article/Supplementary Material, further inquiries can be directed to the corresponding authors.
